# Supplementary material for: The interactome of intact mitochondria by cross-linking mass spectrometry provides evidence for coexisting respiratory supercomplexes
Source: Mol Cell Proteomics. 2017 Dec 8;17(2):216–32. doi: 10.1074/mcp.RA117.000470 (PMC5795388; doi:10.1074/mcp.RA117.000470)
Supplement: Supplemental Data [file supp_RA117.000470_133922_0_supp_23978_fzffwf.zip › spectra_annotation/mito_DR_spectra_annotation/255-1-2-1-1-1.pdf]

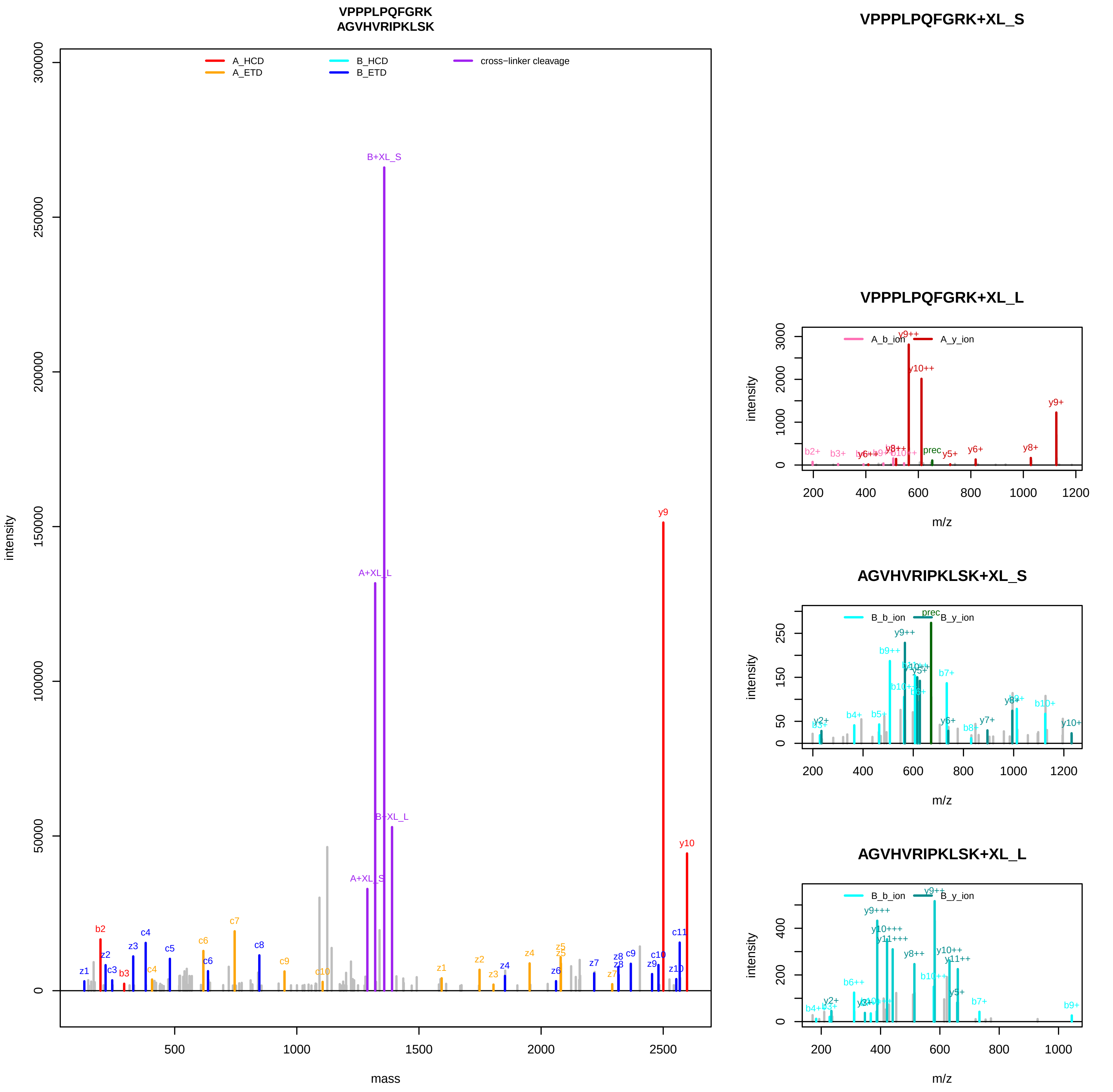
VPPPLPQFGRK
AGVHVRIPKLSK
A_HCD
A_ETD
B_HCD
B_ETD
cross−linker cleavage
intensity
mass
B+XL_S
A+XL_L
B+XL_L
A+XL_S
y9
y10
b2
b3
z1
z2
c3
z3
c4
c4
c5
c6
c6
c7
c8
c9
c10
z1
z2
z3
z4
z4
z5
z5
z6
z7
z7
z8
z8
c9
z9
c10
z10
c11
0
50000
100000
150000
200000
250000
300000
500
1000
1500
2000
2500
VPPPLPQFGRK+XL_S
VPPPLPQFGRK+XL_L
A_b_ion
A_y_ion
y9++
y10++
y9+
y8+
y6+
y5+
prec
b2+
b3+
m/z
AGVHVRIPKLSK+XL_S
B_b_ion
B_y_ion
prec
y9++
b9++
b7+
b10+
y10+
y7+
y6+
b8+
b4+
b5+
y2+
b3+
AGVHVRIPKLSK+XL_L
y9++
y9+++
y10+++
y11+++
y8++
y10++
y11++
b6++
b10++
y5+
b7+
b9+
b4+
b3+
y2+
